# Opinions de femmes sur la législation relative à l’avortement dans la ville de Yaoundé

**DOI:** 10.11604/pamj.2022.43.88.32858

**Published:** 2022-10-19

**Authors:** Josiane Ngo Mayack

**Affiliations:** 1 Institut de Formation et de Recherche Démographiques (IFORD), Yaoundé, Cameroun

**Keywords:** Avortement, Cameroun, loi, perceptions sociales, femmes, Abortion, Cameroon, law, social perceptions, women

## Abstract

**Introduction:**

l'accès à l'interruption volontaire de grossesse (IVG) est restrictif au Cameroun. La législation autorise l'acte dans deux cas: lorsque la grossesse résulte d'un viol ou lorsqu'elle fait courir à la femme enceinte un péril grave pour sa santé. L'avortement pratiqué hors de ce cadre légal est qualifié d'illicite. Cet article identifie et analyse les opinions de femmes vivant dans la capitale Yaoundé, sur les dispositions juridiques nationales relatives à l'IVG.

**Méthodes:**

trente-cinq (35) entretiens semi-directifs ont été réalisés à Yaoundé auprès de femmes âgées de 19 à 40 ans. Les participantes à l'étude recrutées au sortir de consultations (service de planification familiale ou santé maternelle et infantile) ont contribué à la sélection d'autres enquêtées par la méthode «boule de neige». Les données collectées ont fait l'objet d'une analyse thématique de contenu.

**Résultats:**

plus de la moitié des enquêtées est défavorable à la dépénalisation de l'avortement. La dimension transgressive associée à cet acte constitue l'argument majeur soutenant le refus d'un éventuel assouplissement de la législation. Le motif de la sécurité sanitaire est avancé dans les opinions encourageant la dépénalisation. La difficulté à se positionner ou le silence de participantes est une illustration du caractère sensible du sujet de l'IVG.

**Conclusion:**

l'avortement dans son essence est porteur de tensions émanant des interactions entre les logiques institutionnelles, les normes du groupe social et les aspirations individuelles. La polarisation des débats est un frein à l'examen de la question de la dépénalisation sous un prisme multidimensionnel.

## Abstract (en)

**Introduction:**

access to abortion is restrictive in Cameroon. The legislation authorizes the act in two cases: when the pregnancy results from a rape or when it puts the pregnant woman at serious risk for her health. Abortion outside this legal framework is considered illegal. This article identifies and analyses the opinions of women living in the capital Yaounde, on national legal provisions relating to abortion.

**Methods:**

thirty-five (35) semi-structured interviews were conducted in Yaounde with women aged 19 to 40. Study participants recruited from consultations (family planning or maternal and child health service) contributed to the selection of other respondents using the snowball method. The data collected were the subject of a thematic analysis of content.

**Results:**

more than half of those surveyed are against decriminalization. The transgressive dimension associated with abortion constitutes the main argument supporting the refusal of a possible relaxation of the legislation. The reason for health security is advanced in the opinions encouraging decriminalization. The difficulty of positioning oneself or the participants silence is an illustration of the sensitive nature of the subject of abortion.

**Conclusion:**

abortion in its essence carries tensions emanating from interactions between institutional logics, social group norms and individual aspirations. The polarization of debates is a brake on the examination of the issue of decriminalization from a multidimensional perspective.

## Introduction

Les lois en matière d'avortement sont prohibitives dans la majorité des pays d'Afrique subsaharienne. Pourtant, plus de deux tiers des 54 Etats membres de l'Union africaine, dont le Cameroun, ont ratifié le protocole de Maputo qui reconnaît l'avortement médicalisé comme un droit reproductif. Ce texte est considéré comme l'instrument juridique régional «le plus progressiste sur l'accès des femmes à l'avortement légal et sûr en Afrique» [1]. Il recommande aux Etats parties, en son article 14 alinéa 2(c), de protéger la santé reproductive des femmes en autorisant notamment l'avortement médicalisé, en cas d'agression sexuelle, viol, inceste et de mise en danger par la grossesse de la santé mentale et physique de la mère ou sa vie ou encore celle du fœtus. L'harmonisation des législations nationales conformément à cette préconisation demeure problématique. Les restrictions légales de l'avortement en Afrique vont de l'interdiction partielle à l'interdiction absolue.

Une proportion élevée de femmes en âge de procréer en Afrique subsaharienne vit dans des pays dotés d'une législation fortement restrictive, soit 45% contre 47% dans ceux appliquant des lois modérément limitatives et 8% dans des Etats aux dispositions juridiques libérales [2]. La plupart des avortements à risque (c'est-à-dire pratiqués par un prestataire non qualifié et/ou dans un environnement où les conditions médicales ne sont pas respectées) est enregistrée en Afrique subsaharienne, d'où le taux de létalité par avortement particulièrement haut. Il est estimé en 2019 à 185 décès maternels pour 100 000 avortements dans cette région, contre 14 et 16 décès respectivement en Asie et en Amérique latine et les Caraïbes [2].

L'interdiction de l'avortement au Cameroun prend sa source dans la loi n° 67-LF-1 du 12 juin 1967 (issue de la philosophie pénale coloniale), révisée et remplacée par la loi n°2016/007 du 12 juillet 2016 portant code pénal. Les sanctions mentionnées dans l'article 337 prévoient à l'encontre d'une femme qui a avorté, une peine d'emprisonnement de quinze jours à un an et/ou une amende de 5.000 à 200.000 FCFA. Les interruptions volontaires de grossesse (IVG) ne sont autorisées qu'en cas de mise en danger par la grossesse, de la santé de la mère, ou d'une grossesse résultant d'un viol. Le niveau de prévalence actuel de l'avortement au Cameroun est peu documenté. Dans des contextes de pénalisation de la pratique, la qualité des données sur l'avortement est variable et peu fiable, en raison notamment de la rareté des statistiques [3] et «leur couverture dans le temps limitée» [4]. La dernière enquête démographique et de santé (EDS) en date au Cameroun, réalisée en 2018, ne fournit pas d'indication sur l'ampleur de la pratique de l'IVG. L'EDS effectuée en 2011 montre que 7% des femmes ayant déjà eu des rapports sexuels ont déclaré avoir recouru à un avortement provoqué, contre 5% en 2004 [5]. La pratique abortive est plus fréquente chez les femmes résidant dans les métropoles de Yaoundé et Douala (14% et 8,5% respectivement en 2011 et 2004). Elle est majoritairement clandestine dans la capitale Yaoundé et fréquente chez les adolescentes [6]. Les complications liées à l'avortement à risque ou clandestin sont l'une des principales causes de décès maternels hospitaliers au Cameroun [7], notamment dans la capitale [8,9]. Le contexte prohibitif de l'IVG induit le recours à des pratiques réalisées dans des conditions dangereuses. Ce qui nous amène à nous intéresser au regard porté par les femmes, non seulement sur l'avortement mais également sur les dispositions juridiques qui l'encadrent. Cet angle d'analyse est peu documenté au Cameroun. La littérature existante sur l'IVG dans ce contexte géographique porte notamment sur sa contribution à la mortalité maternelle [7-9], les risques sanitaires ou les complications des pratiques clandestines ou à risque [10,11] les déterminants du recours à l'avortement [6,12], les méthodes utilisées [10,11] et la connaissance de la législation en vigueur [12-15].

Les travaux de recherche s'intéressant à la législation interrogent l'effectivité ou la faisabilité de l'accès aux services sécurisés ou médicalisés d'avortement dans un environnement restrictif [13-15]. Le présent article a pour objectif de mettre en lumière les perceptions de la prohibition par les femmes. L'intérêt de cet angle d'étude est de fournir une lecture de la réappropriation individuelle du consensus juridique sur l'interdiction de l'avortement. Dans un contexte de mondialisation où la question de la légalisation de l'avortement suscite la polémique, quels sont les positionnements à un niveau local, vis-à-vis d'une éventuelle dépénalisation? Cet article propose une analyse d'arguments individuels avancés dans le débat sur l'IVG dans un cadre sociétal et juridique réprobateur et répressif.

## Méthodes

**Type d'étude:** les données présentées et discutées sont issues de l'étude qualitative menée dans le cadre de notre recherche doctorale, soutenue en 2017, sur l'utilisation de la pilule contraceptive d'urgence (PCU) dans la capitale camerounaise Yaoundé.

**Population de l'étude:** l'échantillon de 35 femmes dont nous tirons les discours analysés dans cet article, constitue une partie de la population d'étude de notre recherche doctorale (comprenant également des prestataires de services de planification familiale et des responsables de programmes de santé de la reproduction). Ce groupe de femmes âgées de 19 à 40 ans est composé de celles ayant déjà utilisé la PCU (n=21) et d'autres qui n'y ont jamais recouru (n=14). Les participantes recrutées à la sortie de consultations en planification familiale ou en santé maternelle et infantile, grâce au personnel de santé, ont permis d'identifier dans leur entourage d'autres enquêtées grâce à la méthode «boule de neige». Celle-ci consiste en la sélection de nouveaux répondants dans l'environnement des personnes faisant déjà partie de la population d'étude. L'exigence de la représentativité de cette population ne s'appliquant pas à la recherche qualitative, l'échantillon a été constitué en s'assurant de la diversification des caractéristiques sociodémographiques des enquêtées et de l'atteinte du point de saturation des données. Les critères de sélection étaient l'âge, le niveau d'instruction, l'occupation/activité principale, le statut matrimonial, l'appartenance religieuse et l'utilisation antérieure de la PCU dans certains cas. La saturation en recherche qualitative renvoie à une situation de redondance des informations provenant de participants supplémentaires à l'étude [16,17]. L'alternance des méthodes/lieux de recrutement des enquêtées, la diversification des caractéristiques sociodémographiques des individus à interroger et le contrôle de la saturation des données sont des procédés que nous avons utilisés pour limiter les biais.

**Collecte et analyse des données:** des entretiens semi-directifs ont été réalisés en Français dans la métropole de Yaoundé de novembre 2013 à mars 2014. C'est la ville qui enregistre la prévalence contraceptive moderne la plus élevée du pays, soit 27% [18]. Les questions contenues dans le guide d'entretien ont porté sur: i) les connaissances, les opinions en matière de contraception et son utilisation; ii) les relations entre les clientes des services de planification familiale et les prestataires de services de santé; iii) les connaissances, les perceptions de la PCU ainsi que ses usages; iv) la visibilité de cette pilule dans l'offre contraceptive. Les points sur l'IVG ont été abordés dans le volet relatif aux perceptions de la PCU. Les interviews enregistrées grâce à un dictaphone et avec l'autorisation de chaque enquêtée ont été complétées par les notes prises durant les discussions. L'analyse thématique de contenu à partir du logiciel NVivo a permis d'examiner les verbatims transcrits.

**Considérations éthiques:** le protocole de l'enquête de terrain relative à notre recherche doctorale a été approuvé par la commission d'éthique de la recherche de l'Institut d'analyse du changement dans l'histoire et les sociétés contemporaines (Iacchos) de l'Université catholique de Louvain (Belgique) et le comité national d'éthique de la recherche pour la santé humaine au Cameroun. Le consentement éclairé écrit de chaque participante à l'enquête qualitative a été demandé avant d'entamer l'interview. Le contenu du formulaire utilisé pour recueillir ce consentement s'articulait autour de la présentation de la recherche doctorale et ses objectifs, ainsi que sur les garanties d'anonymat, de confidentialité et de libre participation des enquêtées.

## Résultats

La plupart des femmes interrogées souscrivent à la qualification pénale de l'avortement. Elles estiment qu'il s'agit d'un acte condamnable. Les qualificatifs «criminel», «irresponsable», «déviant» sont notamment utilisés pour justifier ce positionnement. Un peu plus des deux tiers de l'échantillon s'opposent à la dépénalisation de l'IVG au Cameroun, trois femmes y sont favorables, trois autres n'ont pas souhaité prendre parti et quatre autres ont refusé de répondre à la question.

**Dépénalisation et crainte de l'augmentation des comportements sexuels à risque:** les réponses défavorables à la dépénalisation de l'avortement peuvent être synthétisées en deux arguments: l'atteinte au droit à la vie et la fuite par les femmes de leurs responsabilités. L'IVG est considérée par les enquêtées concernées par cette section, comme un acte criminel; il s'agit de femmes chrétiennes ou musulmanes. Selon les répondantes qui l'ont invoqué, le deuxième argument consiste à considérer la dépénalisation de l'avortement comme un facteur favorisant la déresponsabilisation des femmes face à la gestion de leur vie reproductive. Certaines répondantes y voient une forme de caution par les responsables politiques, de comportements sexuels à risque.

*«Ce n'est pas une bonne chose parce que ça va encourager certaines filles à ne pas prendre les précautions pour éviter la grossesse. Il faut assumer les risques qu'on prend quand on commence à avoir des rapports»*. (Femme de 23 ans, célibataire, niveau d'instruction primaire). *«Je ne peux pas accepter qu'on change la loi. Tu as des femmes qui font n'importe quoi, elles multiplient les relations. Si vraiment on légalise l'avortement dans ce pays ça risque d'être le désordre. Je ne pense pas que ces femmes-là vont changer leurs comportements»*. (Femme de 28 ans, mariée, niveau d'instruction supérieur).

Les femmes sont particulièrement mises en cause lorsqu'est avancé l'argument de la déresponsabilisation. La responsabilité des hommes est très peu voire pas du tout évoquée par des enquêtées lorsqu'est abordée la question de l'avortement. Cette opinion genrée exclut la participation masculine à la décision d'interrompre une grossesse. Cela s'explique notamment chez les femmes interrogées par les perceptions variables de la responsabilité en matière sexuelle et contraceptive, l'attitude du partenaire/conjoint masculin vis-à-vis de la contraception, le type d'union ou le statut matrimonial, l'existence ou non d'un projet parental au sein du couple.

**La dépénalisation comme garantie à l'accès aux avortements sûrs:** les trois enquêtées qui prennent parti pour une éventuelle légalisation de l'avortement au Cameroun invoquent l'argument sanitaire. Il s'agit d'assurer aux femmes un accès légal et élargi à une IVG sécurisée.

*«Pourquoi ne pas légaliser ça? […] Il y a des jeunes filles qui vont avorter en cachette, en utilisant des procédés douteux qui vont nuire à leur santé. Ça peut même les tuer. […] Ce serait bien qu'on revoit cette loi parce que les femmes risquent leur vie en allant avorter n'importe où»*. (Femme de 29 ans, célibataire, niveau d'instruction supérieur).

Aucune des répondantes favorables à la dépénalisation n'a déclaré avoir des antécédents d'IVG. Leurs positions sur l'assouplissement de la loi se justifient par le problème social et de santé publique que représentent les avortements clandestins et leurs corollaires au Cameroun. Ces enquêtées fondent leur avis sur l'importance d'un meilleur encadrement de la pratique de l'IVG et surtout d'un meilleur accompagnement des femmes qui souhaitent y recourir.

*«Si on libéralise ça fera que les gens iront facilement vers le personnel de santé, ils seront mieux informés et ils auront une bonne prise en charge»*. (Femme de 32 ans, mariée, niveau d'instruction supérieur).

Une des enquêtées insiste sur la relation de confiance qui devrait exister entre le personnel de santé et les femmes qui souhaiteraient interrompre une grossesse. Un changement législatif impliquerait non seulement une adaptation de l'offre de soins et services de santé, mais aussi une communication en direction de l'attitude des prestataires de services de santé. Ceux-ci, compte tenu de la condamnation pénale, religieuse, morale et sociale de l'avortement au Cameroun, de leur propre rapport à ce dernier, peuvent constituer un obstacle à l'accès aux services médicalisés.

**L'indécision sur le sujet:** trois enquêtées n'ont pas énoncé leur prise de position sur la légalisation de l'avortement. Elles conviennent qu'il s'agit d'un sujet sensible pour lequel il est difficile de donner un avis.

*«Cette loi a ses avantages et ses inconvénients. Toute chose a ses bons et ses mauvais côtés. Ce n'est pas évident pour moi de dire si je suis totalement pour ou totalement contre»*. (Femme de 25 ans célibataire, niveau d'instruction supérieur).

Enquêter sur l'avortement dans un environnement sociojuridique prohibitif c'est travailler en terrain sensible. Recueillir les opinions individuelles est un exercice complexe en raison notamment de la suspicion que suscite l'intérêt du chercheur pour un tel sujet et de la pratique du silence autour du recours à la pratique abortive. Ce dernier étant assimilé par des femmes interrogées à un crime, un acte honteux, il se développe une sorte de culture du secret qui crée l'opacité autour du sujet. Dans le cas d'espèce, exprimer un avis favorable à la dépénalisation de l'avortement constitue pour une répondante une prise de risque.

*«Si c'est une légalisation bien surveillée, je suis d'accord. Il faut bien surveiller bien encadrer les choses. Les femmes ont le droit de faire leurs choix. Mais le problème c'est que dans notre société, je ne pense pas que ce genre de loi va arriver. Si je dis à quelqu'un ici que je voudrais qu'on change cette loi il ne va pas me comprendre, il va me juger. C'est difficile parfois de dire vraiment ce qu'on pense. Mais en même temps avorter c'est quand même quelque chose de grave. Donc quelque part je suis aussi d'accord avec ceux qui condamnent ça»*. (Femme de 30 ans, célibataire, niveau d'instruction secondaire).

La difficulté pour une enquêtée à se prononcer sur le sujet s'explique parfois par son expérience personnelle d'une IVG.

*«Avorter ce n'est pas une décision qui est facile à prendre, je l'ai fait. Moi je trouve que chacun a ses raisons. […] Si tu interdis ça arrange certains, si tu n'interdis pas ça arrange aussi d'autres personnes. Pour moi c'est difficile de dire qu'il faut interdire ou ne pas interdire. Comme j'ai dit ce n'est pas facile de choisir d'avorter. Il y a plusieurs situations qui font que c'est ta seule solution comme il y a aussi d'autres femmes qui ont de mauvais comportements et après elles veulent enlever la grossesse. […] Il y a beaucoup de situations vraiment, moi je ne peux pas dire s'il faut interdire ou non»*. (Femme de 25 ans, célibataire, niveau d'instruction primaire).

Cette répondante insiste d'une part sur les difficultés qui entourent la prise de décision et d'autre part sur la diversité des motifs d'avortement. Elle souligne l'importance de ne pas se limiter à l'acte abortif en lui-même et souhaite que la situation personnelle de la femme qui y recourt soit également prise en compte.

## Discussion

La prépondérance de réponses défavorables à la dépénalisation de l'IVG que nous avons soulignée est un résultat similaire à celui obtenu dans une étude réalisée en 2018 dans la ville de Buéa au Cameroun [12]. La plupart des participantes à cette étude s'oppose à la légalisation de l'avortement, même si certaines y ont déjà recouru. Les motifs avancés sont relatifs à la crainte de l'augmentation des comportements sexuels à risques et de leurs corollaires. 23% des femmes interrogées se sont prononcées en faveur de la légalisation. La prohibition sociojuridique de l'avortement au Cameroun s'accompagne d'une stigmatisation à l'encontre des femmes qui y recourent. Cela provient notamment des représentations sociales négatives de l'acte. Celles susmentionnées dans les discours d'enquêtées sont similaires aux perceptions identifiées dans des études réalisées au Bénin [19], au Burkina Faso [20] ou en Zambie [21]. Les aspects relatifs à la morale occupent une place prépondérante dans cette sphère représentationnelle. Ils sont rattachés à la norme officielle (la loi) qui constitue un référentiel en termes de bonnes pratiques. Les opinions en marge de ce référentiel sont considérées comme socialement inappropriées. D'où la difficulté dans le débat sur l'IVG à « parvenir à retenir l'attention autrement que par sa dimension transgressive » [22]. Cela concourt à la polarisation des opinions, sous-tendue notamment par le facteur religieux.

Les autorités religieuses jouent un rôle non négligeable dans le traitement de sujets relatifs à l'intimité sexuelle et reproductive au Cameroun. La ratification du protocole de Maputo par l'Etat a suscité la polémique et fait l'objet de protestations notamment par l'Eglise catholique et des fidèles [23,24]. L'argument de la religion évoqué par des participantes à notre enquête est également avancé par des femmes interrogées dans le cadre d'une étude sur le recours à l'avortement à Lomé au Togo [25].

La pratique d'avortements clandestins, la condamnation sociojuridique de l'IVG et la réprobation morale ou religieuse sont des éléments constitutifs du caractère sensible de l'avortement au Cameroun. Ce caractère expliquerait les hésitations ou le mutisme manifestés par des enquêtées au moment de partager leurs opinions sur le sujet. Des auteurs ont examiné la question du silence qui entoure l'avortement. Ce silence vise, pour les personnes qui en usent, à se prémunir du jugement d'autrui (les proches, le groupe/la communauté d'appartenance) ou de sanctions sociales en raison de la violation du consensus sociojuridique [26-28].

Des recherches antérieures disponibles sur les opinions relatives à la libéralisation de l'avortement en Afrique concernent surtout les professionnels de la santé. Certains au Rwanda pensent que la dépénalisation de l'IVG irait à l'encontre des normes sociétales du pays et de leurs croyances religieuses [29]. D'autres estiment que la suppression des restrictions contribuerait à modifier ces normes qui représentent parfois un obstacle à l'accès et l'utilisation de la contraception. Cependant, des études montrent que l'application d'une loi libérale dans des pays tels que l'Afrique du Sud et la Zambie n'implique pas forcément un changement de mentalités. Les femmes continuent de recourir à des avortements clandestins, à cause notamment de la persistance de la stigmatisation et des sanctions sociales [30,31]. En République démocratique du Congo, des professionnels de la santé préconisent au-delà de l'assouplissement de la loi pour garantir des services sûrs et accessibles en matière d'avortement, le combat de la stigmatisation à travers la mise en œuvre des dispositions du protocole de Maputo et la sensibilisation aussi bien des décideurs politiques, des prestataires de services de santé que des populations [32]. Il faut noter que la fourniture de services médicalisés d'IVG n'est pas un acquis, surtout dans un environnement de défaillance du système de santé. Cet élément constitue un argument en défaveur de l'assouplissement de la loi [33].

Bien que l'inclusion des soins après avortement dans la politique nationale de santé de la reproduction au Cameroun soit une avancée, cette mesure appréhende le problème en aval puisqu'elle porte sur les conséquences sanitaires des pratiques abortives à risque. Un certain nombre de démarches incombe aux femmes avant de pouvoir disposer de services et de soins légaux. L'aboutissement de ces démarches suscite des interrogations dans la mesure où il subsiste dans la loi, des imprécisions liées à l'octroi de l'autorisation d'avorter, aux motifs de l'interruption de la grossesse et aux personnes habilitées à y procéder [13]. La femme est tenue de se justifier et de justifier la prestation médicale sollicitée. Ce qui est susceptible de favoriser des attitudes stigmatisantes pouvant dissuader de recourir au service médicalisé.

Une enquête sur l'avortement dans les métropoles de Yaoundé et Douala a consacré un volet sur le regard porté par les femmes sur le caractère clandestin du recours à l'avortement. Il en ressort que la loi n'exerce pas sur elles une force de persuasion dans la mesure où pour 64% d'entre elles, c'est l'observance des normes sociales, des valeurs traditionnelles relatives à la sexualité et la fécondité qui prime [34]. Vingt-cinq pour cent (25%) déclarent que la peur de la loi est à l'origine de pratiques clandestines. La décision de recourir à l'avortement dépendra des circonstances entourant la grossesse et des intérêts en jeu; les logiques personnelles et les logiques institutionnelles sont mises aux prises.

**Limites de l'étude:** la recherche qualitative ne visant pas la représentativité statistique, les résultats de notre étude ne peuvent pas être extrapolés à l'ensemble de la population de femmes vivant à Yaoundé. Une enquête auprès d'autorités politiques, judiciaires, sanitaires et religieuses et d'une population d'hommes sur le cadre légal de l'avortement permettrait d'effectuer une analyse comparative d'opinions provenant de différents groupes de population. Cela pourrait être fait dans les différentes régions du pays afin de disposer d'une vue globale et détaillée des perceptions de la législation.

## Conclusion

Le caractère illégal de l'avortement dans notre contexte d'étude fait de lui un sujet sensible. Les opinions sur la législation témoignent de la prégnance de la morale dans les perceptions de l'IVG. Les constructions sociales en matière d'interruption de grossesse gravitent autour d'un système de références qui adopte une vision binaire du débat: morale versus transgression. Or, la question d'une éventuelle modification de la loi est multidimensionnelle au regard des implications aussi bien sur le plan du droit, de la santé publique, qu'au niveau politique et social.

### Etat des connaissances sur le sujet

- Les déterminants de l'IVG au Cameroun;
- Les conséquences des avortements à risque ou clandestins au Cameroun;
- La condamnation sociojuridique des pratiques abortives; l'état des lieux de la connaissance de la législation.

### Contribution de notre étude à la connaissance

- Une analyse des opinions sur la législation à partir de l'hypothèse de la dépénalisation de l'IVG au Cameroun;
- Une analyse du rapport individuel à l'avortement.

## Remerciements

L'auteure remercie la commission d'éthique de la recherche de l'Institut d'analyse du changement dans l'histoire et les sociétés contemporaines de l'Université catholique de Louvain (Belgique), le comité national d'éthique de la recherche pour la santé humaine au Cameroun, les autorités sanitaires ayant approuvé la réalisation du recueil d'informations auprès de prestataires de services de planification familiale et les participants à l'étude.
